# Upper Limb Rehabilitation Tools in Virtual Reality Based on Haptic and 3D Spatial Recognition Analysis: A Pilot Study

**DOI:** 10.3390/s21082790

**Published:** 2021-04-15

**Authors:** Eun Bin Kim, Songee Kim, Onseok Lee

**Affiliations:** 1 Department of Software Convergence, Graduate School, Soonchunhyang University, 22, Soonchunhyang-ro, Asan City 31538, Chungnam-do, Korea; ebkim0608@sch.ac.kr; 2 Department of Medical IT Engineering, College of Medical Sciences, Soonchunhyang University, 22, Soonchunhyang-ro, Asan City 31538, Chungnam-do, Korea; songee159@korea.ac.kr

**Keywords:** haptics, stroke, virtual reality, upper limb, rehabilitation, kinematics

## Abstract

With aging, cerebrovascular diseases can occur more often. Stroke cases involve hemiplegia, which causes difficulties in performing activities of daily living. Existing rehabilitation treatments are based on the subjective evaluation of the therapist as the need for non-contact care arises; it is necessary to develop a system that can self-rehabilitate and offer objective analysis. Therefore, we developed rehabilitation tools that enable self-rehabilitation exercises in a virtual space based on haptics. Thirty adults without neurological damage were trained five times in a virtual environment, and the time, number of collisions, and coordinates were digitized and stored in real time. An analysis of variance (ANOVA) of the time and distance similarity changes revealed that as the number of rounds increased, no changes or increases occurred (*p* ≥ 0.05), and the collisions and paths were stable as the training progressed (*p* < 0.05). ANOVA showed a high correlation (0.90) with a decrease in the number of crashes and time required. It was meaningful to users when performing rehabilitation training more than four times and significantly impacted the analysis. This study analyzed the upper limb and cognitive rehabilitation of able-boded people in three-dimensional space in a virtual environment; the performance difficulty could be controlled through variations in rehabilitation models.

## 1. Introduction

Paralysis is a state in which the nerves innervating the muscles lose their function, without changes in the form, resulting in limb numbness or the inability to move. Paralysis can be divided into sensory paralysis and motor paralysis, which occur when there is a disorder in any part of the exercise path from the motor center of the brain to the peripheral nerve and muscle fiber. Upper limb paralysis can result from different causes of brain damage, of which stroke is the most common [1]. Fifty percent of stroke survivors remain impaired, and most patients suffer from hemiplegia [2,3,4,5]. Typical clinical symptoms after stroke include hemiplegia and problems with activities of daily living (ADLs) [6,7]. Minor dysfunction limits one’s daily life, negatively affecting the quality of life with after-effects, including depression [8]. Furthermore, the recovery from these effects is slow and difficult. Previous studies have reported that the upper limb function is decreased in recovered patients [9,10]. Post-stroke patients experience severe stress owing to the gap between previous independence and health and the current dependence on others for ADLs. Thus, mediation is required to provide psychological stability and enable physical rehabilitation [11,12,13]. Existing upper limb rehabilitation methods must be implemented repeatedly over a long period, and rehabilitation is expensive and time-consuming. Furthermore, the treatment is based on a subjective evaluation by a therapist [14,15]. Stroke patients are evaluated by a neurological physiotherapist in clinical practice, and rehabilitation is performed using a proprioceptive neuromuscular facilitation, BOBOTH, Brunnstrom, or Rood approach [16,17]. However, under-experienced therapists cannot analyze a patient’s condition and are unable to receive and process feedback from therapists and patients [18]. These problems have led to the development of low-cost, high-efficiency rehabilitation treatments using digital media, such as virtual reality (VR) and augmented reality [19,20]. Clinical rehabilitation using robots has been shown to be efficacious in both acute and chronic patients, and improves function and daily movements [21]. A rehabilitation treatment system developed for objective and quantitative evaluation training using VR showed that tactile and vibration feedback improved neuroplasticity [22,23,24,25,26]. Haptic, a human-computer interaction (HCI) technology, provides users with information acquired by touch in virtual, augmented, and real environments [27,28]. The device can present virtual forces; therefore, it can be used by physical and occupational therapists for rehabilitation therapy. The haptic is capable of active resistance in the active range of motion mode. This provides the minimum amount of force necessary to satisfy the prescribed motion, which can be resisted by the user’s opposing force, thereby increasing the muscle strength, and increasing the coordination of the eyes and arm muscles. Therefore, it can be applied in rehabilitation [26,29].

Specifically, haptic and virtual reality-based rehabilitation therapy maximizes the user’s sense of immersion within a low-cost and safe environment, allowing users to perform self-rehabilitation exercises [30,31]. In addition, haptic-based systems can be used for rehabilitation based on the Rood approach. Rood therapy, which provides adequate stimulation and enables reflexive motor induction, makes motor engram acquisition possible [32]. It also creates indelible memories through a simulation (promotion) and activates the brain through thousands of iterations, based on neuroplasticity theory [33,34,35].

**Figure 1:**
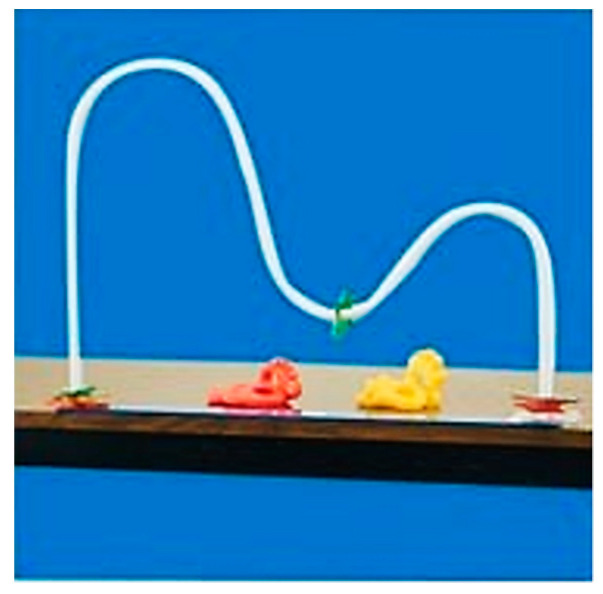
Traditional rehabilitation; double-curved shoulder arc.

Range of motion (ROM) is a method used by clinicians to check the health condition of the joint and improve the range of motion, affecting the qualitative characteristics of active motion [36]. The double-curved shoulder arc (Fit to Function Therapy, NY, USA) (http://newsite.fittofunctiontherapy.com (accessed on 1 April 2021)) form (Figure 1) used in upper limb rehabilitation is a rehabilitation tool that can check active movement through abnormal conversion behavior and increase the performance difficulty through multiple transformations [37,38]. However, tools that can be analyzed objectively analyzed during the rehabilitation in a virtual environment are currently being developed. Therefore, there is an urgent need for non-contact diagnosis and rehabilitation to expand the medical accessibility, and research on the development of rehabilitation tools capable of self-rehabilitation exercise is urgently needed [39,40]. This work seeks to develop rehabilitation tools that enable self-rehabilitation training and analysis through haptics via rendering and a rehabilitation model modified by a double-curved shoulder arc form to the virtual environment. It was intended to conduct basic research with able-bodied people to verify the reliability of the tool and present appropriate criteria through analysis and methods that could identify the process of rehabilitation change.

## 2. Materials and Methods

### 2.1. System Design

#### 2.1.1. Haptics

The coaching system, used video-based motion recognition and motion tracking, and provided subject feedback. Haptic is a coaching device used for virtual reality or remote rehabilitation and is detected by a sensor [41]. In addition, the two-way force provides feedback, and a vibration and texture are used to maximize the user’s senses [42].

**Figure 2:**
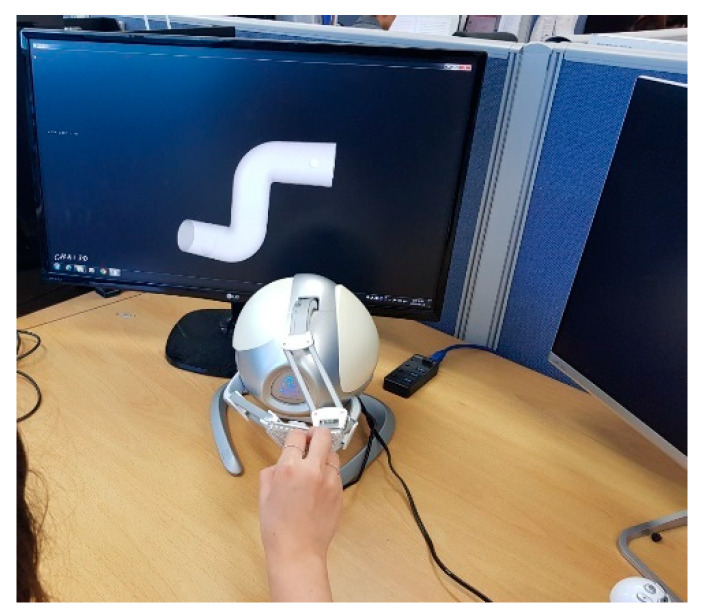
Upper limb rehabilitated by catching the haptic probe and passing through the three-dimensional rehabilitation model.

In this study, a three degrees of freedom (3DOF) haptic device (3D Touch Controllers, Novint Falcon, NM, USA) for translational motion was used. There are a maximum of 3DOF joint motions in the body [43,44]. The user was moved using a haptic probe. In this case, *x*, *y*, and *z* coordinates were obtained in real time, and the user’s position was tracked within a three-dimensional (3D) virtual space using the OpenHaptics-based Chai3D API written in C++. A 3D model rendered in a virtual space creates a mathematical model that can be reproduced in space. A 3D object is represented by a 3D line through the rendering process and can have a texture and volume similar to those of a real object. The 3D model was created using the Auto Desk Inventor (Professional 2016, Autodesk, CA, USA) (Figure 2).

**Figure 3:**
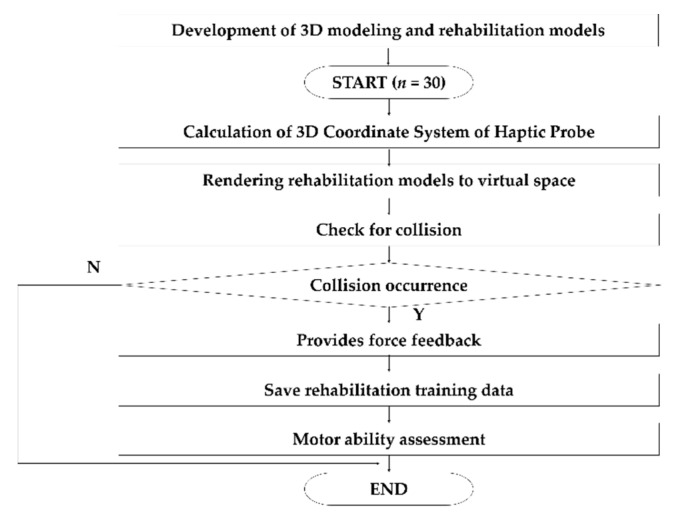
Design scheme of the proess to assess motor ability by providing force feedback and storing collision data within the model during rehabilitation in real time.

Based on the algorithm presented in Figure 3, the model was developed to calculate and provide feedback on the forces occurring in the virtual space when the haptic interaction point of the haptic probe, which is controlled by the user, collides through the movement inside the rehabilitation model through the haptic probe. The system was designed to count the number of crashes that occurred off the normal travel path. By measuring the time from the entrance of the rehabilitation model to the exit, we received feedback on the time taken for rehabilitation, identified the pathways, and visualized the shortest distance through the model. The obtained coordinates were used to graph the paths in 2D and 3D using MATLAB (Matrix Laboratory MathWorks 2018, MathWorks, MA, USA).

#### 2.1.2. Pre-Processing

Three preprocessing steps were applied to identify changes in performance through the location coordination of the rehabilitation system, which was applied five times: (i) a principal component analysis (PCA); (ii) normalization; and (iii) warping.

High-dimensional data processing makes it difficult to properly express the meaning of the data owing to the exponential increase in observation and memory usage, which is called the curse of dimensionality [45]. A PCA best describes changes in multivariate analysis data based on an eigenvector in statistics to return high-dimensional data to low-dimensional data (dimensionality reduction) [46,47,48]. In this study, the covariance matrix Σ was obtained by Equation (1) for raw data A, which are x, y, and z values stored in real time. Here, T is the linear transformation, and n is the number of columns. Subsequently, the eigenvalues λ and eigenvector α of the covariance matrix were calculated and internalized with the original data X to extract the characteristics that appeared during the rehabilitation while preserving the three-dimensional data as much as possible.

(1) $$\Sigma =cov\left(X\right)=\frac{1}{n}AA^{T}$$

Subsequently, the category of the data was transformed by Equation (2) into [0, 1] to minimize data duplication and reduce its impact on the relative size [49,50].

(2) $$Normalization\left(A_{New}\right)=\frac{A-A_{min}}{A_{max}-A_{min}}$$

The lengths of the normalized datasets were bound to vary from one person to another. A 1:1 convergence is difficult when vectors differ in length; therefore, the sequence of the two time series to be compared was intended to be matched. Here, $X=\left(x_{1},\;x_{2},\cdots ,x_{m}\right)$ and $Y=\left(y_{1},\;y_{2},\cdots ,y_{n}\right)$ were defined, the Euclidean distances were obtained, and the warped vector was obtained through Equations (3) and (4) [51].

(3) $$W=w_{1},\;w_{2},\cdots ,w_{m}max\left(m,n\right)<K<m+m-1$$

(4) $$Warped\;vector\left(x,\;y\right)=min\left(\sqrt{\overset{k}{\underset{i=1}{\sum }}w_{n}/n}\right)$$

### 2.2. Evaluation

In this study, the Euclidean distance (ED) and Manhattan distance (MD) were used to analyze the similarity through the distance between two vectors expressed in n-dimensions to observe the difference from the first results of the same person after using the rehabilitation system five times. In addition, we analyzed the similarity with the dynamic time warping (DTW) method, which is a distance-based similarity measurement commonly used in a time series. It is also intended to analyze whether the two vectors have a linear relationship through cosine similarity (CS) and correlation coefficient (CC), which are methods of measuring the similarity based on the angles that they form. The similarities were analyzed using MATLAB software. We wanted to verify the hypothesis by setting it as null and void the idea that the change in performance will increase or be equal to the number of applications. The confidence interval of the ANOVA was set at 95% using Minitab software (version 19, Minitab, Inc., State College, PA, USA); it was used to verify the hypothesis of the distance similarity analyzed earlier and the significant difference between the number of collisions and the time required in the experiments conducted by 30 subjects. Tukey’s post hoc test was used to analyze the correlation between the number of crashes and the time required to complete the course.

#### 2.2.1. Subjects

This study was conducted after receiving approval from the Research Ethics Review Committee(1040875-202009-BM-068) and 10 min of prior explanation of experiments, consent descriptions, etc., to the participants. Experiments were performed five times every 30 s and took a total of 15 min. A total of 30 healthy people in their 20 s (22.6 ± 2.37 years) who were not diagnosed with neurological impairment participated in this study. Before starting the training program, the participants were verbally instructed to move from the entrance to the exit. The time taken to complete the course and crash records were not mentioned.

#### 2.2.2. Euclidean Distance

The ED, a measure well suited to the meaning of the actual distance, is a measure of the shortest direct route election between two vectors using Equation (5) [52,53].

(5) $$ED=\sqrt{\overset{k}{\underset{i=1}{\sum }}{\left(y_{i}-x_{i}\right)}^{2}}$$

#### 2.2.3. Manhattan Distance

The MD is also called a taxicab geometry and is the concept of substituting coordinates for cities. In other words, the idea is that, in real cities, when moving from the start point to the arrival point, the shortest distance must be recalculated based on the roads and any blocks because they are not accessible, similar to calculating ED [53]. These expressions are given by Equation (6):

(6) $$MD=\overset{k}{\underset{i=1}{\sum }}\left|x_{i}-y_{i}\right|$$

#### 2.2.4. Dynamic Time Warping

DTW is used to compare the similarities between two time-series patterns at different speeds [54]. By matching the positions between two time-series datasets, DTW can be flexibly compared and properly matched against distorted vectors [55,56]. For two time-series graphs, each m, n in length, i.e., $X=\left(x_{1},\;x_{2},\cdots ,x_{m}\right)$ and $Y=\left(y_{1},\;y_{2},\cdots ,y_{n}\right)$, distance d of two vectors is calculated as ${\left(x_{i}-y_{j}\right)}^{2}$ and filled using a warping matrix. Subsequently, to find the smallest among the previous values, and to determine the warping path, the optimal path navigation is determined through Equation (7), which is centered on the value at the top right.

(7) $$\begin{array}{c} DTW\left(1,\;1\right)=d\left(1,\;1\right)\\ DTW\left(1,\;j\right)=d\left(1,\;j\right)+d\left(1,\;k-1\right),\;2\leq j\leq n\\ DTW\left(i,1\right)=d\left(i,\;1\right)+d\left(i-1,\;1\right),\;2\leq i\leq m\\ DTW\left(i,\;j\right)=d\left(i,\;j\right)+min\{\begin{array}{c} D\left(i-1,\;j-1\right)\\ D\left(i-1,j\right)\\ D\left(i,j-1\right) \end{array},\;2\leq j\leq n,\;2\leq i\leq m \end{array}$$

#### 2.2.5. Cosine Similarity

The CS is the similarity of two vectors, which can be obtained using the cosine angle between two vectors, with a value of 1 if the two vectors are in the same direction and −1 if it is opposite, and the closer the value is to 1, the higher the similarity [57]. The expression of the cosine similarity between the two vectors $X=\left(x_{1},\;x_{2},\cdots ,x_{m}\right),\;Y=\left(y_{1},\;y_{2},\cdots ,y_{n}\right)$ is the same as in Equation (8).

(8) $$CS\left(\theta \right)=\frac{X\cdot Y}{\left\|X\|\|Y\right\|}=\frac{\sum_{i=1}^{k}X_{i}\cdot Y_{i}}{\sqrt{\sum_{i=1}^{k}{\left(X_{i}\right)}^{2}}\cdot \sqrt{\sum_{i=1}^{k}{\left(Y_{i}\right)}^{2}}}$$

#### 2.2.6. Correlation Coefficient

The CC, an indicator of the degree and direction of the relationship between variables, is expressed as a value between −1 and 1. The closer the value is to zero, the lower the correlation, and the closer it is to −1 or 1, the higher the correlation. A static correlation of the direction of increase or decrease is defined as +, and a negative correlation between the direction of increase or decrease is defined as − [58,59]. When there are two time-series graphs $X=\left(x_{1},\;x_{2},\cdots ,x_{m}\right)$ and $Y=\left(y_{1},\;y_{2},\cdots ,y_{n}\right)$, the correlation coefficient is given by Equation (9).

(9) $$CC=\frac{\sum_{i=1}^{k}\left(x_{i}-\bar{x}\right)\left(y_{i}-\bar{y}\right)}{\sqrt{\sum_{i=1}^{k}{\left(x_{i}-\bar{x}\right)}^{2}}\cdot \sqrt{\sum_{i=1}^{k}{\left(y_{i}-\bar{y}\right)}^{2}}}$$

## 3. Results

Datasets preprocessed using PCA were preserved at an average rate of approximately 99.7%.

### 3.1. Visuality

#### 3.1.1. Time Taken and the Number of Collisions

**Figure 4:**
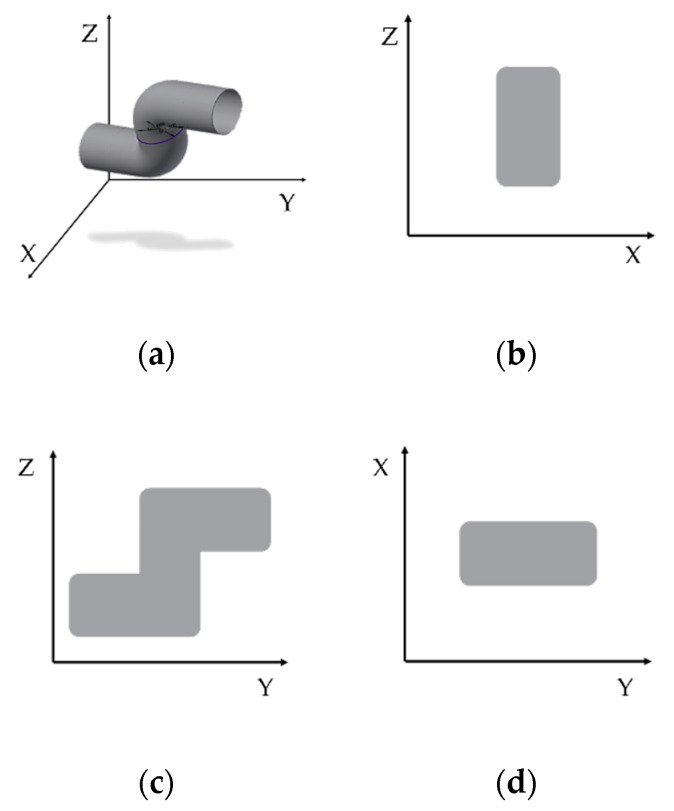
(**a**) Three-dimensional rehabilitation model rendered in virtual space, and view of the model along the (**b**) XY axis, (**c**) YZ axis, and (**d**) XZ axis in a two-dimensional plane.

**Figure 5:**
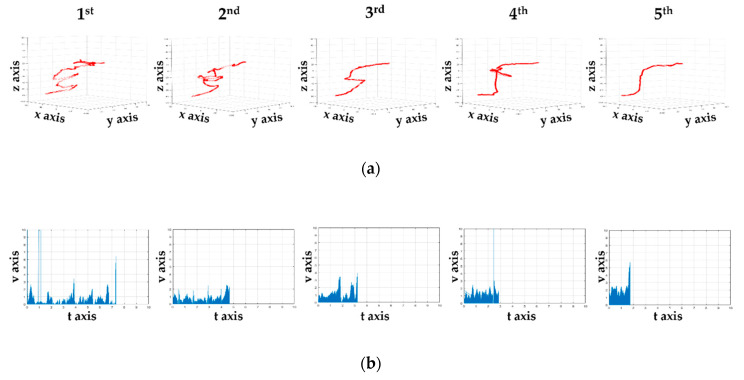
Three-dimensional paths of the probe and sequentially displayed time vector graphs: (**a**) 3D paths of the probes in turn and (**b**) sequentially displayed time vector graphs. The 3D path graphs shows the collision points and movements. The collision and time decrease as the rehabilitation progresses.

**Figure 6:**
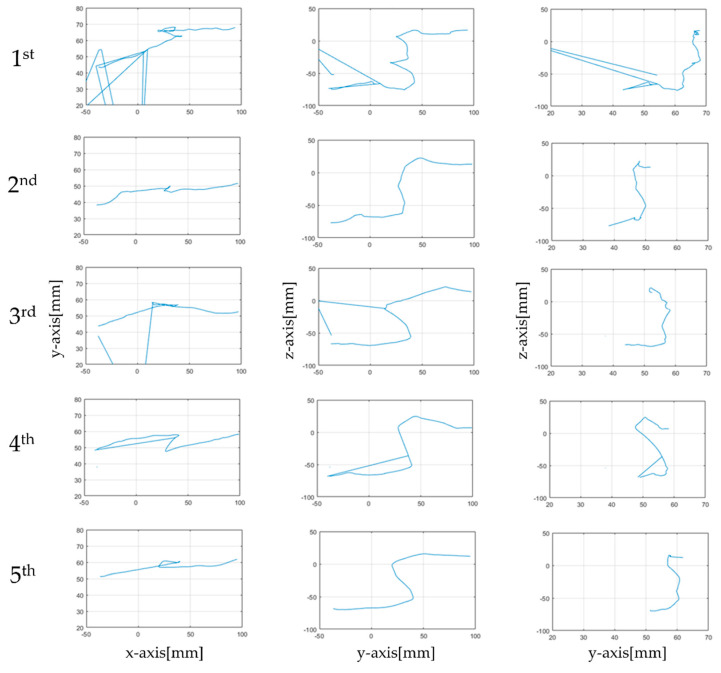
These graphs are a 2D representations of the paths of movement stored in three dimenScheme.

Figure 4a shows the treatment model in a 3D virtual space. The data expressed at this time were raw data without preprocessing. In Figure 4b, the XZ axis identifies the *x*-axis travel path of the haptic probe. Figure 4c shows the YZ axis used to check the path of travel along the *y*-axis, and Figure 4d shows the YX axis used to check the path of the *z*-axis. Figure 5 and Figure 6 show the data obtained from five rehabilitation training sessions of one of the subjects sequentially from the top. Figure 5a shows the movement coordinates of the probe on a 3D graph, providing a visual indication of the path. Using a haptic probe, hand tremors causing collisions and difficult movements were captured when passing through the inside of the model. In addition, the gap shown in the 3D graph was narrow when it was difficult to move the probe using the upper limbs. In addition, as the sessions progressed, the gap between the sessions was greater than the first, as more rapid movements became possible. Figure 5b shows vector values over time. The vector value of the section in all time axes stabilized as the rotation progressed.

Figure 6 shows the path of the *x*, *y*, and *z*-axes identified by the directions of the arrows and numbers indicated in Figure 4. Figure 5 shows representative sequential rehabilitation data. The *x*-axis of the probe in the first row, the *y*-axis of the probe in the second row, and the *z*-axis in the third row are identified. Movements were unstable until the third rehabilitation session, and the direction changed dramatically and tended to be overly skewed in the on-direction. However, the number of crashes in four and five rehabilitation sessions decreased with repeated learning.

#### 3.1.2. Similarity

In the pre-processed dataset, the *x*-coordinates can show the characteristics of tremors in which the subject approaches or moves away from the subject in the model when applying the system. The *y*-coordinates are from the side of the moving position from start to end, and the *z*-coordinates can show the characteristics of the upward and downward tremors in the model.

Table 1 shows a descriptive statistics comparing the similarity between the first performance and the second, third, fourth, and fifth time series. In the case of distance similarity, it can be seen that the average distance of the *x*-axis is greater than that of the *y*-axis, for which it is believed to be more difficult to recognize the depth of the space than the height. In addition, for an angle-based similarity, the average was high (*x* avg.: 0.95, *y* avg.: 0.97, *z* avg.: 0.98), and the correlation was strongly positive (*x* avg.: 0.77, *y* avg.: 0.87, *z* avg.: 0.98) [60], indicating the relevance of the two time series.

### 3.2. Statistics

#### 3.2.1. Time Taken and the Number of Collisions

For the number of collisions showing significant differences (*p* = 0.00), there was a large difference in the number of collisions (Table 2). The results of post-analyses conducted to verify the significant differences in the number of collisions between the first few rounds compared to the first found showed significant differences from the fourth to the fifth time series, with an average 2.85-fold decrease (Table 3).

Comparison and analysis of the mean at each time point showed no significant differences (*p* = 0.32) (Table 4). However, the correlation between the change in the number of timescale collisions and the mean change in time was 0.09, and the time required decreased as the number of crashes decreased (Table 5).

#### 3.2.2. Similarity

**Figure 7:**
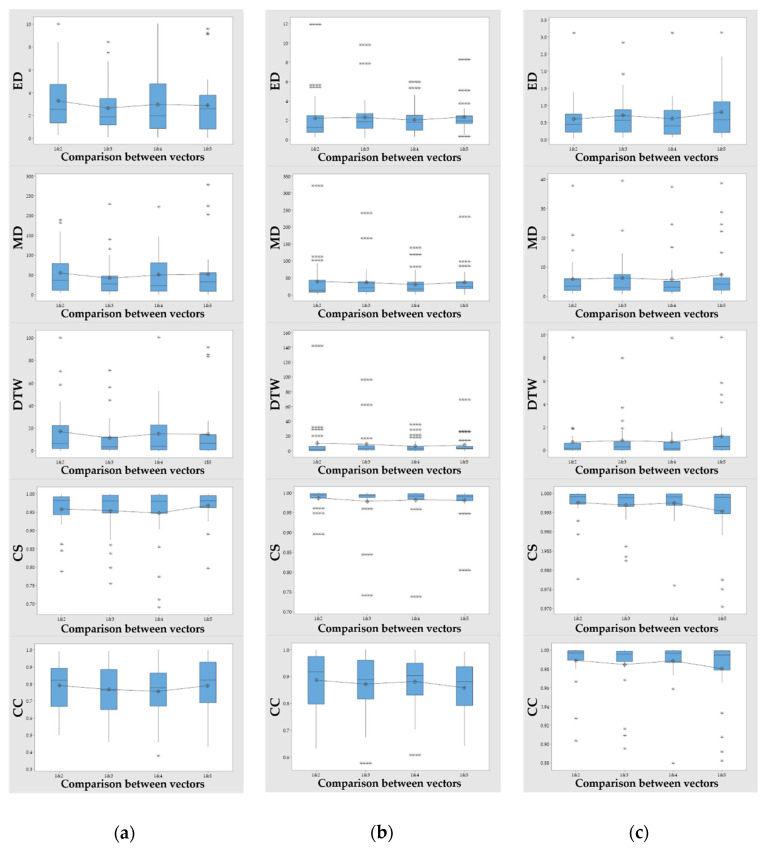
Analysis of the similarity between the two time series vectors, ED, MD, DTW, CS and CC in turn: (**a**) *x*, (**b**) *y*, (**c**) *z* axes.

Figure 7 shows a box plot of the ANOVA results for ED, MD, DTW, CS and CC (*p* > 0.05). The *x*-axis requires spatial recognition horizontal to the ground, whereas the scatter size of the *y*-axis, which indicates the total travel path in space, and that of the *z*-axis, which requires vertical spatial recognition, shows that the perceived tremor values are concentrated and that the deviation of the target is insignificant.

## 4. Discussion

Motion paralysis occurs when there is a disorder in any part of the movement path from the motor center of the brain to the peripheral nerves and muscle fibers. Among them, upper limb paralysis is the most common disorder after a stroke, for which various treatment methods have been proposed, including rehabilitation exercise therapy through initial collaboration with rehabilitation medicine to prevent future effects and help relieve paralysis symptoms and restore motor function through repeated rehabilitation exercises [36]. However, the existing rehabilitation exercise was conducted through a subjective judgement of the occupational therapists. Thus, it is problematic when quantitative and objective evaluations are not carried out. In addition, the need for non-contact diagnosis and treatment has emerged to deal with the financial burden of repeated and lengthy rehabilitation training [39,40]. Therefore, rehabilitation of psychological and motor therapy through digital media, such as virtual reality and augmented reality, is emerging [37].

Virtual reality allows the input and output of information from various objects in a three-dimensional virtual space, and reverse-tactile information helps to realistically recognize various environments and increase user immersion. Moreover, virtual reality technology is in the spotlight for applications in rehabilitation because it can create individual environments and perceptual stimuli that motivate patients while preserving the potential to interact with real environments, including the patients [38]. However, the spatial cognitive abilities of virtual reality also require time for able-bodied adults to adapt to multiple activities. In addition, the cognitive ability and analysis methods for three-dimensional space and the evaluation criteria for various rehabilitation tools are ambiguous; therefore, basic research on each tool is urgently needed. In this study, rehabilitation tools and analysis methods for analyzing haptic-based active upper extremity movements were presented, and basic research was conducted by analyzing cognitive abilities in the three-dimensional space of able-bodied adults.

In this study, a total of five rehabilitation evaluations were conducted using subjects in their 20 s (22.6 ± 2.37 years) with no neurological damage at intervals of 30 s. Subsequently, the hypothesis was verified after visualizing the change in the time and number of collisions and the distance similarity using the data stored in real time.

As shown in Figure 5a and Figure 6, the tremors on the paths of *x*, *y*, and *z* were significantly reduced in the fifth round compared with the first round. At this point, the *x*-axis shows the movement of the haptic probe of the subject inside and outside of the target, the *y*-axis shows the path from beginning to end, and the *z*-axis shows the characteristics when the haptic probe moves up and down. The movement in and out of a space indicates that the depth of the space is level with the ground and differs from subject to subject based on use. Conversely, the *z*-axis shows the difference between vertical surfaces within a space. It can be interpreted that the abnormal values shown in Figure 7a,c are caused by the lack of recognition of the depth of space. The *y*-axis with which the path of movement can be viewed is ideal to move from left to right, but if reversed by a collision during rehabilitation, if the space of the curve is not understood and if the understanding of the time of termination is insufficient, a zigzag path will appear. Thus, as shown in Figure 7b, there were more anomalies along the *x*-axis. The interpretation of the *x*, *y*, and *z*-axes is also associated with hypothesis verification. As the number of uses increases, the cognitive skills of the user in the space are improved, particularly when rehabilitation tools are applied more than four times. Over time, however, the null hypothesis was not rejected, resulting in a significant or equal change in time. This means that there was time variability in each subject of the study. The results in Figure 5b indicate that, although the time appears to decrease, this is not the case for many of the subjects studied. Finally, it can be concluded that the spatial cognitive ability of the subjects is not directly related to time, but the correlation results in a reduction in the number of tremors and collisions from the fourth round or higher.

In addition, the number of collisions can be described as a result of a change in distance similarity depending on the number of rounds compared to the beginning, which is the result of a tremor. An example of the change in the distance of a vector shows that the electron has a large rate of change in its distance between each point, compared with two vectors having the same start and end points and two vectors having the same shaking condition. As a result of this study, the null hypothesis that there will be the same large difference between the two vectors was adopted in Figure 7; although the mean changes in the *x*, *y*, and *z*-axes were not constant, the mean increased compared to the first and fourth rounds on the *x*-axis, meaning that there would be a significant change in the spatial recognition of the inside and outside when the rehabilitation was repeated four times. The *y*-axis decreased until four iterations and then increased after five iterations. In addition, given that the path was different from the first time when was repeated five times, the opposite situation on the ideal path was reduced by the fifth times, which means that the opposite situation (zigzag movement) on the ideal path was reduced, and the values were concentrated owing to the small spread. Finally, as the results of the *z*-axis indicate, the mean increased when compared with the first and third rounds and the first and fifth rounds, which means that there was a significant change in the spatial cognitive abilities above and below when the process was repeated three times and five times. In conclusion, the changes in the number of collisions, time, and distance similarity can produce meaningful results in at least four training sessions and can be analyzed.

In this study, compared to subjective evaluations of therapeutic performance by rehabilitation specialists and rehabilitation therapists, experimental measurements of the resistance, track speed, and location error due to unnecessary forces allow for the digitalization of data for more objective performance and treatment evaluations. Existing rehabilitation methods have shown that physical therapy conducted based on a therapist’s subjective judgment can be analyzed and evaluated objectively through haptic-based rehabilitation receptors called muscle spindles and Golgi tendon organs [61]. Therefore, they can be used as a treatment to reduce muscle tension and enhance stability through haptic-based stretching movements. In addition, physical therapy, such as strong stimulation and tendon compression, is applied to the affected area, which stimulates the Pacinian corpuscle, thus reducing muscle tension [62]. This study will allow therapists to correct patient misbehavior and provide feedback on repetitive activities. In addition, visual, auditory, and olfactory stimulation of the brain nerves relieves myopia tension and enhances stability, integrates the senses, and restores function [63]. Moreover, virtual reality movements are shown on a screen to express the image of the haptic probe moving on with its own on, and problems are solved by interacting with the tasks that appear on the screen, creating user motivation [64]. In this study, the patients were able to recognize their physical abilities objectively, understand the progress of their recovery, and evaluate their visible physical abilities.

## 5. Conclusions

Based on the double-curved shoulder arc form, which is rehabilitation tool to check an active movement, we developed a tool to analyze self-rehabilitation exercise through haptic device and rehabilitation change processes. As a basic study analyzing the three-dimensional cognitive ability of the rehabilitation environment applied in a virtual world by selecting an able-bodied person as a target, it was possible to visually identify the changes in time, number of collisions, and distance similarity according to the rotation, and it was found that a meaningful analysis is possible only after at least four training sessions. The results of this study indicate that, the level of difficulty in terms of performance is thought to be controllable through a future transformation of the rehabilitation model, and the approach can be used in occupational therapy. In addition, as a basis for the Rood approach, it is possible to provide repetitive feedback and accumulate data accordingly, which can be utilized as big data. Furthermore, the system will be used in clinical and rehabilitation sites to manipulate the perceived use, perceived case of use variables in the technology acceptance model, and external variables to analyze the interaction with technology, user attitudes, and behavior.

## Figures and Tables

**Table 1:** Mean and standard deviation of the similarity verification results between time series.

Warped Vectors	Similarity	*x*	*y*	*z*
1 and 2	ED	3.27 ± 2.54	2.24 ± 2.34	0.59 ± 0.59
	MD	54.81 ± 53.76	39.20 ± 62.20	5.92 ± 7.48
	DTW	17.02 ± 23.88	10.31 ± 26.35	0.72 ± 1.78
	CS	0.95 ± 0.04	0.98 ± 0.02	0.99 ± 0.00
	CC	0.79 ± 0.13	0.88 ± 0.10	0.98 ± 0.02
1 and 3	ED	2.66 ± 2.10	2.30 ± 2.07	0.70 ± 0.61
	MD	41.70 ± 49.14	36.13 ± 50.00	6.29 ± 7.86
	DTW	11.37 ± 17.41	9.25 ± 19.96	0.86 ± 1.58
	CS	0.94 ± 0.06	0.97 ± 0.50	0.99 ± 0.00
	CC	0.75 ± 0.13	0.87 ± 0.10	0.98 ± 0.02
1 and 4	ED	2.98 ± 2.54	2.04 ± 1.48	0.61 ± 0.61
	MD	50.00 ± 55.20	30.84 ± 33.54	5.71 ± 7.75
	DTW	15.13 ± 22.46	6.31 ± 8.79	0.73 ± 1.77
	CS	0.94 ± 0.08	0.98 ± 0.04	0.99 ± 0.00
	CC	0.75 ± 0.15	0.88 ± 0.09	0.98 ± 0.02
1 and 5	ED	2.88 ± 2.58	2.35 ± 1.55	0.08 ± 0.77
	MD	51.60 ± 67.50	36.50 ± 42.66	7.35 ± 9.21
	DTW	14.74 ± 25.34	7.90 ± 13.14	1.23 ± 2.19
	CS	0.96 ± 0.04	0.98 ± 0.03	0.99 ± 0.00
	CC	0.79 ± 0.15	0.85 ± 0.09	0.98 ± 0.03

**Table 2:** Differences in the number of collisions that occurred during a rehabilitation process depending on the sequence in which they were conducted.

	df	F
Between Groups	4	6.47 *
Within Groups	145	
	149	

* *p* < 0.05.

**Table 3:** Results of post-analysis analyzed by round when looking at the difference in the number of collisions per route; there was a significant difference in the fourth and fifth rounds compared to the first.

Rounds (I)	Rounds (J)	Mean Difference (I − J)
1	2	0.56
	3	1.60
	4	2.73 *
	5	3.00 *
2	1	−0.56
	3	1.03
	4	2.16 *
	5	2.43 *
3	1	−1.60
	2	−1.03
	4	1.13
	5	1.14
4	1	−2.73 *
	2	−2.16 *
	3	−1.13
	5	0.26
5	1	−3.00 *
	2	−2.43 *
	3	−1.40
	4	−0.26

* p < 0.05.

**Table 4:** There was no significant difference in the time spent conducting rehabilitation by round.

	df	F
Between Groups	4	1.17
Within Groups	145	
	149	

**Table 5:** Average changes in the number of collisions and time spent were highly correlated, indicating that more than four rehabilitation attempts are recommended.

		Time Spent	Number of Collisions
Time spent	Correlation coefficient	1	0.90 *
Number of collisions	Correlation coefficient	0.90 *	1

* *p* < 0.05.

## Data Availability

Not applicable.
